# Dynamics of dual-orbit rotations of nanoparticles induced by spin–orbit coupling

**DOI:** 10.1515/nanoph-2024-0586

**Published:** 2025-01-24

**Authors:** Yu Zhang, Qian Lin, Zikuan Zhuang, Fei Lin, Ling Hong, Zhen Che, Linqing Zhuo, Yongyao Li, Li Zhang, Dongxu Zhao

**Affiliations:** Guangdong-Hong Kong-Macao Joint Laboratory for Intelligent Micro-Nano Optoelectronic Technology, Foshan, Guangdong 528225, China; School of Physics and Optoelectronic Engineering, 47868 Foshan University, Foshan, Guangdong 528225, China; Guangdong Science and Technology Infrastructure Center, Guangzhou, Guangdong 510033, China; School of Electronics and Information, Guangdong Polytechnic Normal University, Guangzhou, Guangdong 510665, China

**Keywords:** spin–orbit coupling, orbital angular momentum, optical manipulation, optical force

## Abstract

Spin–orbit coupling (SOC) in tightly focused optical fields offers a powerful mechanism for manipulating the complex motion of particles. However, to date, such a mechanism has only been applied to the single-orbit motion for particles, while multi-orbital dynamics have not yet been experimentally demonstrated. Here, the theoretical and experimental realization of dual-orbit rotational dynamics of nanoparticles in a tightly focused circularly polarized Laguerre-Gaussian beam is reported. Analyses reveal that the dual-orbit rotation of nanoparticles originates from SOC in a tightly focused vortex beam, with the motion velocity and direction determined by the topological charge of the beam. Experimentally, the dual-orbit rotation of polystyrene nanoparticles was observed for the first time using an inverted optical tweezer. In addition, the rotation velocity showed a clear linear dependence on the topological charge of the incident beam. This work reveals the pivotal role of SOC in enabling precise dual-orbit control at the nanoscale, paving the way for applications in optical sorting, grinding and delivery of microparticles.

## 1 Introduction

Optical force, arising from the exchange of momentum during the interaction between light and matter, has been instrumental over the past half-century in enabling the trapping and manipulation of micro-/nano-scale objects [1], [2], [3], [4], [5]. Pioneered by Ashkin in 1970, optical trapping technology, known as optical tweezers, has been recognized as the optimal method for manipulating the movement of microscopic matter across biomedical, physical chemistry, and micro-optomechanics fields due to its non-contact, non-destructive, and high-precision manipulation capabilities [6], [7], [8], [9], [10]. Traditional Gaussian beam-based optical tweezers rely on the gradient force to confine particles to the focal region, enabling precise positioning and translational motion. While this technique is highly effective for single-particle manipulation, its reliance on a single optical beam inherently limits its ability to perform multi-degree-of-freedom manipulation of multiple particles. Recent advancements have extended the functionality of optical tweezers by incorporating optical fields with other external techniques to overcome previous limitations. For instance, through the coupling of adaptive multiphysical fields, a single laser beam can now enable the simultaneous manipulation of multiple particles with multi-degree-of-freedom capabilities, including translation, rotation, and immobilization [11]. Additionally, dynamic rotation of bio-micromotors has been achieved using annular scanning traps [12], [13], while hydrodynamic effects have been leveraged for collaborative multi-particle manipulation [14], [15].

In addition to these advancements, it has been demonstrated that manipulating the degrees of freedom of the optical field, particularly by controlling optical angular momentum (AM), multi-degree-of-freedom particle manipulation can also be achieved [16], [17], [18], [19], [20], [21]. Typically, the spin angular momentum (SAM) of light is related to its polarization, while the orbital angular momentum (OAM) is associated with the helical wavefront structure of the light [22], [23], [24]. Compared with traditional Gaussian beams, the manipulation of AM has provided unprecedented flexibility and stability in the trapping and manipulation of nanoscale objects, showing significant potential in applications such as optical motors [16], optical wrenches [25], optical grinders [26] and so on. Due to its unique helical wavefront structure, additional AM can be imparted to the trapped particles by the light fields carrying OAM, allowing them to rotate around the beam axis under the drive of the beam. This capability has garnered widespread research interest in recent years [27], [28], [29], [30], [31], [32], [33], [34], [35]. However, OAM-based optical manipulation studies are typically limited to single-orbit motion [28], [29], [30], except that a few works realize multi-orbit manipulation of multiple particles using six-fold symmetric nanoparticle array [25], counter-rotating Laguerre-Gaussian (LG) modes [26], focused vector vortex beams carrying radial-splitting optical chirality [31], local AM in annular fields without intrinsic OAM [33], or customized structured light fields [34], [35]. Although these approaches offer new avenues for dual-orbit manipulation, they are typically limited to specific types of particles (such as chiral or Janus particles) [31], [33] or rely on structured light fields, thus limiting their broader applicability.

The spin–orbit coupling (SOC) of light, enabling interaction and conversion between SAM and OAM, provides a novel approach for regulating AM and potentially achieving multi-orbit particle motion using only a simple light field [36], [37], [38], [39], [40], [41], [42], [43], [44], [45]. Recently, the SOC effect in waveguide optical tweezers has been theoretically predicted, where particles rotate clockwise and counterclockwise along the two sides of a ring-shaped waveguide under the action of circularly polarized light [46]. However, it is constrained by its reliance on waveguides and absorptive particles and has not yet been experimentally verified. On the other hand, the SOC effect in tightly focused optical fields has been experimentally confirmed to induce the orbital motion of nanoparticles [40], [47], [48], but it is typically limited to single-orbit motion, with few experimental studies on the more complex dual-orbit rotational motion.

In this paper, the dual-orbit rotational dynamics of nanoparticles induced by SOC in a tightly focused light field are demonstrated both theoretically and experimentally. We reexamine the theoretical model of a tightly focused circularly polarized LG beam and its OAM density distribution. Numerical simulations are performed to calculate the optical forces acting on a non-absorptive polystyrene (PS) nanoparticle, which predict the dual-orbit rotation of nanoparticles. Experimentally, PS nanoparticles are employed as probes to visualize the SOC effect, and the dual-orbit rotation of nanoparticles induced by SOC is observed for the first time. By adjusting the magnitude and sign of the topological charge of the incident light, the rotation velocity and direction of the nanoparticle can be flexibly controlled. A good linear relationship is found between the rotation velocity and the topological charge. Our findings not only offer a novel technique for multi-degree-of-freedom manipulating nanoparticles but also provide a new approach to experimentally visualize the SOC effect, laying the groundwork for future explorations in controlling optical AM in nanoscale systems.

## 2 Theory and formulation

To reveal the physical origin of the SOC of light, the theoretical model is established using a circularly polarized LG beam. In general, the complex amplitude of a conventional unfocused circularly polarized LG beam in polar coordinates can be expressed as [49]

(1) $$\begin{array}{ll} E_{nm}\left(r,\phi ,z=0\right) & ={\left(\frac{\sqrt{2}r}{w_{0}}\right)}^{m}L_{n}^{m}\left(\frac{2r^{2}}{w_{0}^{2}}\right)\\ & \;\times \exp\left(-\frac{r^{2}}{w_{0}^{2}}+im\phi \right)\hat{e} \end{array}$$

where *r* and *ϕ* are the radial distance and azimuth angle in cylindrical coordinates, respectively. *w*_0_ is the Gaussian beam waist, $L_{n}^{m}\left(x\right)$ is an adjoint Laguerre polynomial, *n* is the radial mode number, and *m* is the topological charge, which defines the helical phase structure of the beam. $\exp\left(im\phi \right)$ is vortex phase factor, and $\hat{e}$ is the unit vector of circularly polarized light. When propagating in free space at an arbitrary distance *z*, the beam complex amplitude takes the form

(2) $$\begin{array}{ll} E_{nm}\left(r,\phi ,z\right) & =C{\left[\frac{r}{w\left(z\right)}\right]}^{m}L_{n}^{m}\left(\frac{2r^{2}}{w^{2}\left(z\right)}\right)\\ & \;\times \exp\left(-\frac{r^{2}}{w^{2}\left(z\right)}-\frac{ikr^{2}}{2R\left(z\right)}\right)\\ & \;\times \exp\left(im\phi -i\left(m+2n+1\right)\zeta \left(z\right)\right)\hat{e} \end{array}$$

here

(3) $$\begin{array}{ll} w\left(z\right) & =w_{0}\sqrt{1+{\left(\frac{z}{z_{R}}\right)}^{2}},R\left(z\right)=z\left[1+{\left(\frac{z_{R}}{z}\right)}^{2}\right],\\ \zeta \left(z\right) & =\arctan\left(\frac{z}{z_{R}}\right) \end{array}$$

*C* is the normalization constant, $z_{R}=kw_{0}^{2}/2$ is the Rayleigh range, *k* = 2π/*λ* is the wave number of light of wavelength *λ*, and $\zeta \left(z\right)$ is the Gouy phase.

For the analysis of the SOC effect of a tightly focused high-order circularly polarized LG beam under strong focusing, according to the Richards–Wolf vector diffraction method [18], [50], the focused field near the focus can be expressed as an integral of plane waves of the form of

(4) $$\begin{array}{ll} & E^{\left(out\right)}\left(r,\phi ,z\right)\\ & \;=\left(\begin{array}{c} E_{x}^{\left(out\right)}\\ E_{y}^{\left(out\right)}\\ E_{z}^{\left(out\right)} \end{array}\right)=\frac{ik\;f\exp\left(-ikf\right)}{2\pi }\overset{\theta_{\max}}{\underset{0}{\int }}d\theta \overset{2\pi }{\underset{0}{\int }}d\phi \\ & \;\times \left[E_{\phi }^{\left(in\right)}\left(\begin{array}{c} \sin\phi \\ -\cos\phi \\ 0 \end{array}\right)+E_{\rho }^{\left(in\right)}\left(\begin{array}{c} \cos\theta \cos\phi \\ \cos\theta \sin\phi \\ \sin\theta \end{array}\right)\right]\\ & \;\times \exp\left(ikr\right)\sin\theta \sqrt{\cos\theta } \end{array}$$

In Eq. (4), $E_{\phi }^{\left(in\right)}$ and $E_{\rho }^{\left(in\right)}$ are the angular and radial components of the incident field on the pupil plane, respectively. *f* is the focal length of the high-NA objective. $\theta_{\max}=\arcsin\left(NA/n_{0}\right)$ is the maximum angle determined by NA, and *n*_0_ is the refractive index in the image space. On the pupil plane, the relationship between the cylindrical coordinate system and the Cartesian coordinate system can be expressed as

(5) $$\left\{\begin{array}{c} e_{\phi }=e_{x}\sin\phi -e_{y}\cos\phi \;\\ e_{\rho }=e_{x}\cos\phi -e_{y}\sin\phi \; \end{array}\right.$$

The two orthogonal base vectors of circularly polarized light can be given in Cartesian coordinates as

(6) $$\left\{\begin{array}{c} e_{L}=e_{x}-ie_{y}\;\\ e_{R}=e_{x}+ie_{y}\; \end{array}\right.$$

Therefore, combining Eqs. (2)–(6), the electric field distribution of circularly polarized light near the focal plane can be obtained as

(7) $$\begin{array}{ll} E_{x}^{\left(out\right)} & =\frac{iAkfe^{-ikf}}{2}\\ & \;\times \left[i^{m\pm 2}I_{m,m\pm 2}{\text{e}}^{\text{i}\left(m\pm 2\right)\phi }+i^{m}J_{m,m}e^{im\phi }\right] \end{array}$$

(8) $$\begin{array}{ll} E_{y}^{\left(out\right)} & =\frac{Akfe^{-ikf}}{2}\\ & \;\times \left[\pm i^{m\pm 2}I_{m,m\pm 2}{\text{e}}^{\text{i}\left(m\pm 2\right)\phi }\mp i^{m}J_{m,m}e^{im\phi }\right] \end{array}$$

(9) $$E_{z}^{\left(out\right)}=iAkfe^{-ikf}\left[i^{m\pm 1}K_{m,m\pm 1}e^{i\left(m\pm 1\right)\phi }\right]$$

In Eqs. (7)–(9), *m* is the topological charge, representing the order of OAM in the circularly polarized LG beams, $A=\sqrt{2P\sqrt{\mu_{0}/\varepsilon_{0}}}/w_{0}$ , *P* is incident power, and *ε*_0_ and *μ*_0_ are the vacuum permittivity and permeability, respectively. The functions *I*(*r*, *z*), *K*(*r*, *z*) and *J*(*r*, *z*) are expressed as

(10) $$\begin{array}{c} I_{u,v}\left(r,z\right)=\overset{\theta_{\max}}{\underset{0}{\int }}u_{v}\left(f\sin\theta \right)J_{u}\left(kr\sin\theta \right)\left(\cos\theta -1\right)\\ \;\times \sin\theta \sqrt{\cos\theta }e^{ikz\cos\theta }d\theta \\ K_{u,v}\left(r,z\right)=\overset{\theta_{\max}}{\underset{0}{\int }}u_{v}\left(f\sin\theta \right)J_{u}\left(kr\sin\theta \right)\left(\cos\theta +1\right)\\ \;\times \sin\theta \sqrt{\cos\theta }e^{ikz\cos\theta }d\theta \\ J_{u,v}\left(r,z\right)=\overset{\theta_{\max}}{\underset{0}{\int }}u_{v}\left(f\sin\theta \right)J_{u}\left(kr\sin\theta \right)\\ \;\times \sin^{2}\theta \sqrt{\cos\theta }e^{ikz\cos\theta }d\theta \end{array}$$

Here, *J*_*u*_ is the *u*th-order Bessel function of the first kind. The magnetic field distribution $H^{\left(out\right)}\left(r,\phi ,z\right)$ near the focus can also be obtained in a similar manner.

It is seen from Eqs. (7)–(9) that if the incident light is a left-handed circularly polarized LG beam, then ring structures corresponding to the OAM orders *m* and *m* − 2 appear simultaneously in the electric field distribution near the focal plane. Conversely, for a right-handed circularly polarized beam, rings associated with topological charges *m* and *m* + 2 emerge simultaneously. Thus, under tight focusing conditions, the SOC effect partially converts the SAM of circularly polarized light into OAM, resulting in an increase of two OAM orders and the formation of an additional ring. This leads to the coexistence of different orbital modes.

After deriving the focusing field, the SAM and OAM distributions in the focal plane of the focused field can be further calculated based on the field distribution at the focal plane. For a harmonic field with an angular frequency *ω*, the time-averaged SAM and OAM densities are defined as

(11) S=ImεE*×E+μH*×H4ωL=ImεE*⋅r×∇E+μH*⋅r×∇H4ω

Moreover, to investigate the mechanism of the interaction between nanoparticle and beam, the optical force acting on the particle should be quantitatively analyzed. Consider a dielectric spherical particle with a radius *R* and a refractive index *n*_*p*_, illuminated by an arbitrary electromagnetic wave characterized by the fields **E**_inc_ and **H**_inc_. The incident wave interacts with the particle, resulting in scattered fields **E**_sca_ and **H**_sca_ outside the particle. Given the known scattered fields **E**_sca_ and **H**_sca_, the time-averaged optical forces $\left\langle F\right\rangle$ acting on the particle can be evaluated by integrating the Maxwell stress tensor over a surface *S* that encloses the particle

(12) $$\left\langle F\right\rangle =\underset{s}{\oint }\left\langle \overset{↔}{T}\cdot \hat{n}ds\right\rangle$$

here $\hat{n}$ is the outwardly directed normal unit vector to the surface and $\overset{↔}{T}$ is the Maxwell stress tensor [4]

(13) $$\overset{↔}{T}=\varepsilon_{1}\text{EE}+\mu_{1}\text{HH}-\frac{1}{2}\left(\varepsilon_{1}E^{2}+\mu_{1}H^{2}\right)\overset{↔}{I}$$

with *ε*_1_ and *μ*_1_ denoting the permittivity and permeability of the medium surrounding the particle, **E** = **E**_inc_ + **E**_sca_ and **H** = **H**_inc_ + **H**_sca_ representing the total fields outside the particle, and $\bar{I}$ is the unit dyadic. Once the incident field is known, the scattered field can be calculated using the finite-difference time-domain (FDTD) algorithm, which we will calculate using 3D-FDTD method (Ansys Lumerical) in the following discussion.

## 3 Results and discussion

**Figure 1:**
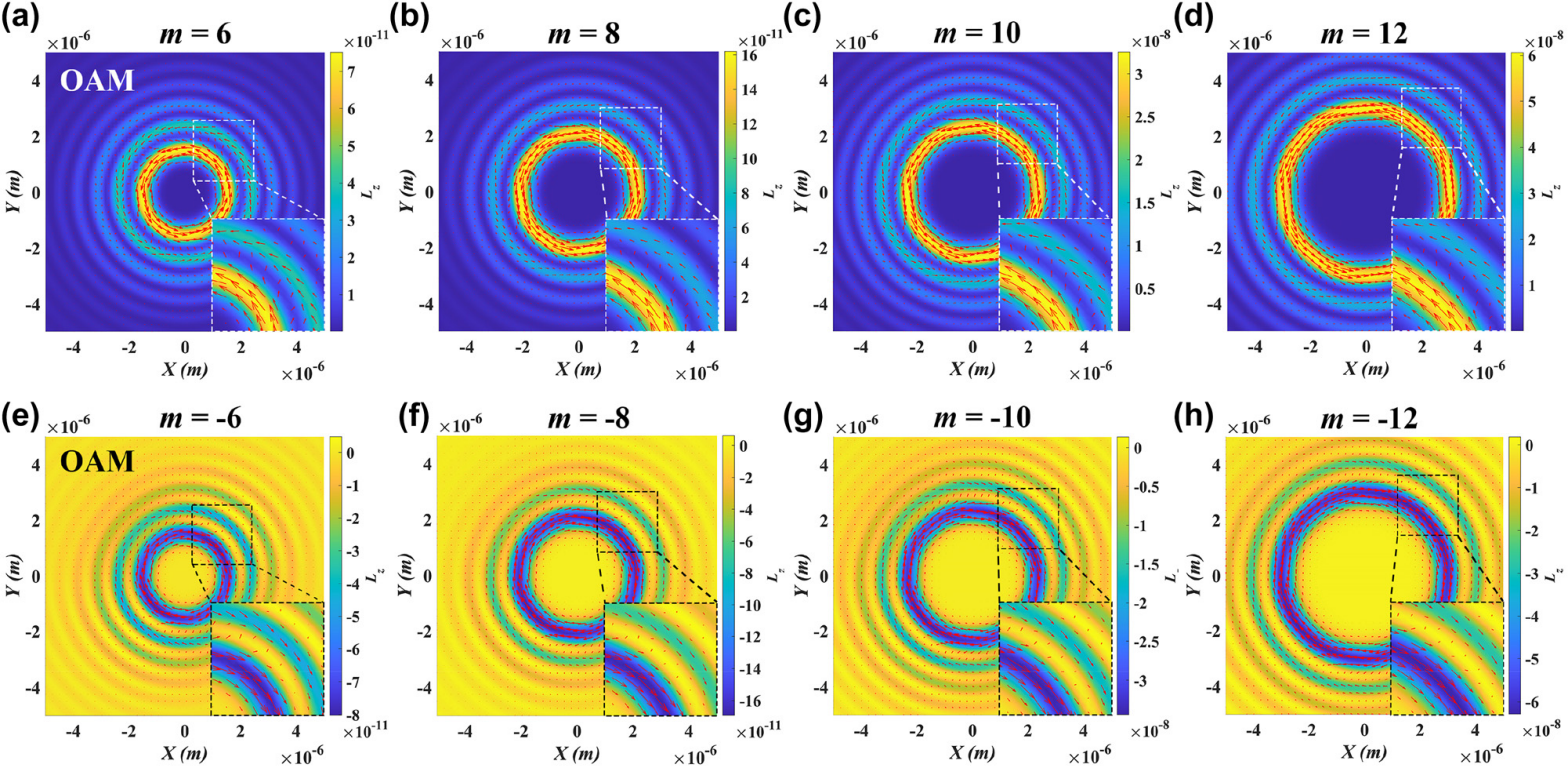
Numerical calculation of the OAM distribution in the focal plane of tightly focused circularly polarized LG beams under different topological charges. (a–d) OAM density distributions and momentum directions for right-handed circularly polarized light at topological charges *m* = 6, 8, 10, 12. The OAM density exhibits a multi-ring structure and there is an obvious secondary bright ring caused by the SOC effect in addition to the central bright ring. The inset provides a magnified view of a selected region, showing that the momentum vector rotates counterclockwise along the orbit. (e–h) For *m* = −6, −8, −10 and −12, the incident light is a left-handed circularly polarized. The overall structure of OAM is similar to that of the positive topological charge case, but the direction of momentum is opposite. The inset presents a magnified area illustrating that, in this case, the momentum vector rotates clockwise along the orbit.

To intuitively understand the SOC effect in a tightly focused circularly polarized LG beam, the OAM density distribution in the focal plane under different topological charges *m* was numerically calculated by using Eq. (11), as shown in Figure 1. In the calculation, the input power is assumed to be *P* = 100 mW and the incident light wavelength in free space is *λ* = 1.064 μm. The Gaussian beam waist *w*_0_ = 1 mm and the input illumination is focused by an objective lens with NA = 1.25. Figure 1(a)–(d) correspond to the OAM distribution of right-handed circularly polarized light with positive topological charges *m* = 6, 8, 10 and 12, respectively. It can be observed that the OAM exhibits a multi-ring structure, with the intensity gradually decreasing from the center outward. The central bright ring arises from the OAM carried by the beam itself, while the secondary bright ring is induced by the SOC effect, converting SAM into OAM. Notably, the OAM distribution features a slightly darker secondary bright ring adjacent to the central bright ring, characterized by Eqs. (7)–(9). This indicates that when the particle size and light intensity are appropriate, dual-orbit dynamics of the particles can be observed in the tightly focused beam. As the topological charges *m* increase, the OAM density strength significantly increases, and the diameter of the rings expands. For *m* = 6, the OAM is concentrated near the center with a smaller ring, as shown in Figure 1(a). As *m* increases to *m* = 12, the ring expands significantly, and the OAM strength becomes more pronounced across the ring, as shown in Figure 1(d). This trend implies that increasing the topological charge enhances both the orbital motion radius and the particle velocity. Additionally, each figure includes an enlarged inset to clearly show the momentum density vectors (red arrows), illustrating the direction of momentum flow in the optical field. For positive topological charges, the momentum rotates counterclockwise along the orbit, causing nanoparticles in the focused field to rotate counterclockwise. In contrast, Figure 1(e)–(h) display the OAM distribution for left-handed circularly polarized light with negative topological charges *m* = −6, −8, −10 and −12, respectively. Although the overall distribution is similar, the OAM direction is reversed, reflecting the opposite helical phase direction of the beam. This reversal is confirmed by the momentum density vectors, which flow clockwise for negative topological charges, as indicated by the red arrow. The theoretical prediction of SAM-to-OAM conversion in a tightly focused circularly polarized LG beam is corroborated by Figure 1, confirms the dynamic properties of OAM in such fields and provides a physical explanation for the dual-orbit rotation mechanism of the particles. By adjusting the magnitude and sign of the topological charge, the intensity and distribution of OAM can be flexibly controlled, thus regulating the velocity and direction of particle orbital motion.

**Figure 2:**
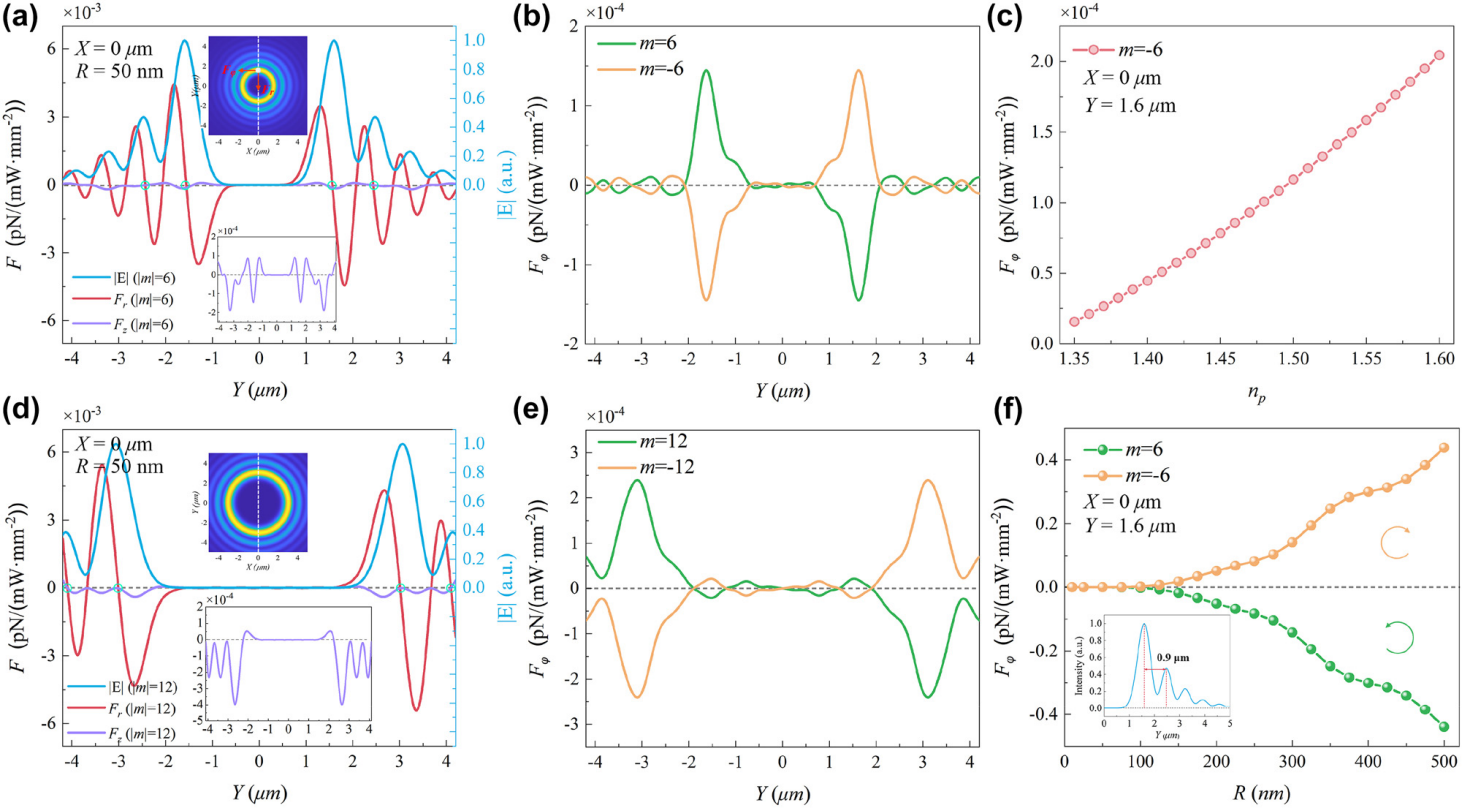
Numerical simulation of normalized optical force acting on a nanoparticle under different topological charges. (a) The normalized optical force profile along the *Y*-direction for a 50 nm radius PS particle at *X* = 0 μm, with topological charges |*m*| = 6. The red curve represents the radial trapping force *F*_*r*_, the purple curve represents the axial scattering force *F*_*z*_, and the blue curve represents the normalized electric field intensity. The top inset illustrates the distribution of the normalized electric field for |*m*| = 6 and the bottom inset shows the magnified view of the axial scattering force *F*_*z*_. (b) The distribution of the tangential driving force *F*_*φ*_ along the *Y*-direction for |*m*| = 6. (c) The dependence of the *F*_*φ*_ on the refractive index of a nanoparticle with a radius of 50 nm for *m* = −6. (d) The normalized optical force along the *Y*-direction for the same 50 nm radius PS particle at *X* = 0 μm, but with topological charges |*m*| = 12. (e) The *F*_*φ*_ distribution along the *Y*-direction for topological charges |*m*| = 12. (f) The variation of *F*_*φ*_ as a function of the nanoparticle radius. The inset shows that the peak interval of the normalized electric field intensity along the *Y*-direction is 0.9 μm when |*m*| = 6.

The 3D-FDTD (Ansys Lumerical) method was performed to quantitatively evaluate the optical force acting on a PS particle immersed in a tightly focused field, as shown in Figure 2. In the simulation, the total calculation space was set as 9 μm × 9 μm × 4 μm, and the PS particle was positioned at the center of the simulation region. The refractive index of PS particles was defined as *n*_*p*_ = 1.5717 at *λ* = 1.064 μm, and the refractive index of the surrounding environment was set to *n*_0_ = 1.33. The electric and magnetic field components, calculated by Eqs. (7)–(9), were imported as the incident light source, and the source size was specified as 5 μm × 5 μm. It is noteworthy that the source is located above the particle and at least one wavelength away from the particle to ensure that the incident wave is fully expanded before propagating to the particle, thereby accurately simulating the interaction between light and particles. Perfectly matched layer (PML) boundary conditions were applied in the *X*, *Y*, and *Z*-directions, and the minimum mesh width of 2 nm was used to accurately solve the total field and calculate the optical force. The optical force on the particle was calculated by integrating the Maxwell stress tensor $\overset{↔}{T}$ over the surface of the particle.

**Figure 3:**
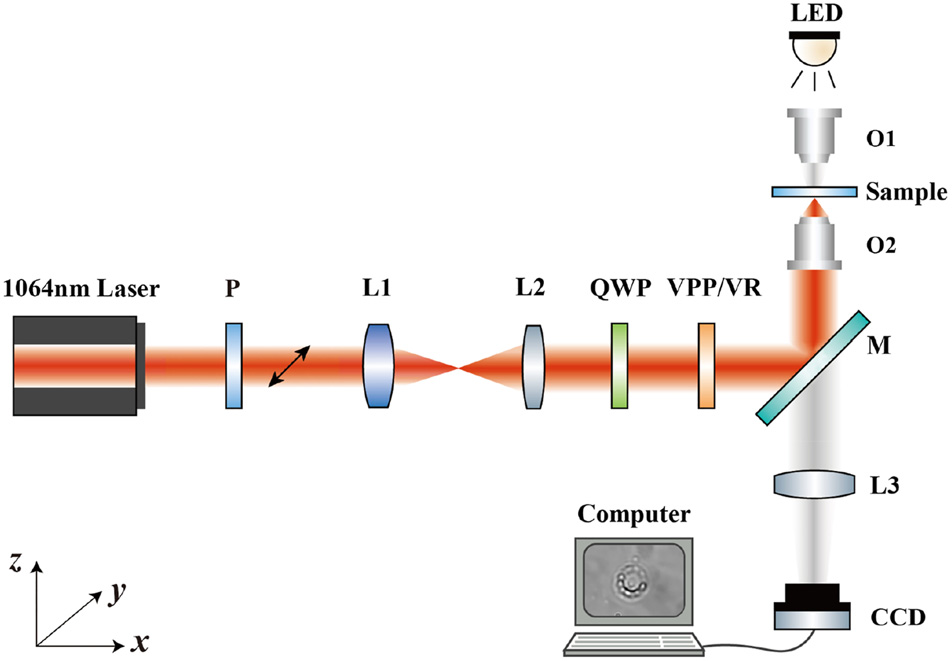
Schematic diagram of the inverted optical tweezers setup for rotating PS nanoparticles. P, polarizer; L1-L3, lenses; QWP, quarter-wave plate; VPP/VR, vortex phase plate/vortex retarder; M, mirror; O1, 10× object; O2, 100× object; CCD, charge coupled device.

We calculated the normalized optical force along the *Y*-direction (*X* = 0 μm) for a 50 nm radius PS particle, as shown in Figure 2(a). In Figure 2(a), the blue curve represents the normalized electric field intensity |*E*| along the *Y*-direction, marked by the dashed line in the top inset. The red curve shows the variation of the radial trapping force *F*_*r*_ along the *Y*-direction. At *X* = 0 μm, the radial trapping force is directed along the *Y*-direction, as indicated by the red arrow in the top inset. From the variations of |*E*| and *F*_*r*_ along the *Y*-direction, multiple equilibrium positions can be identified on both sides of the optical field. Among them, the trapping positions corresponding to the central bright ring and the adjacent secondary bright ring are found at *Y*_1_ = −2.4 μm, *Y*_2_ = 2.4 μm, *Y*_3_ = −1.6 μm, and *Y*_4_ = 1.6 μm, as shown by the cyan circles in the figure. Thus, the radial trapping force *F*_*r*_ confines the PS nanoparticle within the optical orbit. Additionally, the axial scattering force *F*_*z*_ acting on the particle along the *Z*-direction is shown as the purple curve, with a magnified view in the bottom inset. The axial scattering force is symmetrically distributed on both sides of the *Y*-direction and is one order of magnitude smaller than the radial trapping force *F*_*r*_. In experiments, the gravitational force of the particle is usually applied to counterbalance this axial scattering force due to the inverted optical tweezer system (see Figure 3), thus stabilizing the PS particle within the focal plane. It is important to note that for topological charges of the same absolute value (such as |*m*| = 6), the distributions (magnitude and direction) of both *F*_*r*_ and *F*_*z*_ along the *Y*-direction remain unchanged. However, the tangential driving force *F*_*φ*_ (which, at *X* = 0 μm, is directed along the *X*-direction) is nonzero at the equilibrium positions and depends strongly on the topological charge. Figure 2(b) illustrates *F*_*φ*_ along the *Y*-direction for topological charges *m* = 6 and *m* = −6. It is evident that for opposite topological charges, *F*_*φ*_ at a given position has the same magnitude but opposite directions. For a given topological charge, positions equidistant from the *Y*-axis center yield *F*_*φ*_ of the same magnitude but in opposite directions. This suggests that when trapped at either of these trapping points, *F*_*φ*_ tends to rotate the PS nanoparticle around the beam axis.

Furthermore, to investigate the influence of the topological charge on the optical force, we examine the normalized optical force along the *Y*-direction for |*m*| = 12, as shown in Figure 2(d) and (e). Figure 2(d) is similar to Figure 2(a), showing the radial trapping force *F*_*r*_ (red curve) and axial scattering force *F*_*z*_ (purple curve) along the *Y*-direction. As the topological charge increases, the peak of the normalized optical force rises, consistent with the increase in OAM density observed in Figure 1. At the same time, the diameter of the ring expands, as shown in the top inset, and the oscillation of the normalized optical force moves further from the center. Similarly, due to the radial trapping force *F*_*r*_, multiple trapping points appear on both sides of the optical field. These trapping positions, corresponding to the central bright ring and the adjacent secondary bright ring, are located at *Y*_5_ = −4.1 μm, *Y*_6_ = 4.1 μm, *Y*_7_ = −3 μm, and *Y*_8_ = 3 μm, as indicated by the cyan circles. Figure 2(e) shows the distribution of the tangential driving force *F*_*φ*_ along the *Y*-direction for a topological charge |*m*| = 12. As with Figure 2(b), *F*_*φ*_ follows the same trend with respect to the sign of topological charge and increases in magnitude as the topological charge grows.

To further explore the universality of the SOC effect on different materials, Figure 2(c) shows the dependence of *F*_*φ*_ on the refractive index *n*_*p*_ of a 50 nm radius nanoparticle. It is seen that when the refractive index increases from 1.35 to 1.6, *F*_*φ*_ rises almost linearly from 0.15 × 10^−4^ to 2 × 10^−4^ pN/mW/μm^−2^. This suggests that the proposed SOC effect can be extended to dielectric particles or biological cells with various refractive indices, provided the optical contrast between the particle and the surrounding medium is suitable. In addition to the refractive index, the size of the particle is also crucial to visualize the SOC effect, especially since biological samples may differ significantly in size. To investigate the influence of particle size on the observed SOC effect, we calculated the variation of the tangential driving force with particle radius, as shown in Figure 2(f). Figure 2(f) illustrates the variation of the tangential driving force *F*_*φ*_ with particle radius *R* when the particle is located at *X* = 0 μm and *Y* = 1.6 μm, with a topological charge of |*m*| = 6. One can find that as the particle radius *R* increases from 10 nm to 500 nm, *F*_*φ*_ gradually increases, and reversing the topological charge reverses the direction of *F*_*φ*_. Although optical forces for various particle sizes can be theoretically obtained, in practice, the particle size is constrained by the OAM density distribution required to detect dual-orbit dynamics induced by the SOC effect. That is, the maximum particle size depends on the gap between the two orbits. Specifically, the inset in Figure 2(c) shows the normalized electric field intensity distribution along the *Y*-direction for |*m*| = 6, revealing a 0.9 μm gap between the two peaks. If the particle diameter exceeds 0.9 μm, the particles on both orbits will influence each other, potentially preventing the observation of dual-orbit motion. Therefore, smaller nanoparticles should be chosen as experimental probes to visualize the SOC effect through dual-orbit dynamics of particles.

We constructed an inverted optical tweezer system to verify the proposed SOC mechanism and optical force, as depicted in Figure 3. A linearly polarized monochromatic continuous wave laser (*λ* = 1,064 nm, 100 mW, Coherent Inc.) serves as the primary light source for the experiment. A polarizer *P* is employed to adjust the laser polarization, producing horizontally polarized light (along the *y*-axis). The beam is then expanded and collimated by a 4*f* system consisting of two lenses: L1 with a focal length of 150 mm and L2 with a focal length of 200 mm. After collimation, the beam passes through a quarter-wave plate (QWP), converting the linearly polarized light into circularly polarized light carrying SAM. The beam is further transformed by a vortex phase plate (VPP), imparting OAM to create a vortex beam. It should be noted that in the experiment, the VPP is used to generate a vortex beam with a topological charge *m* ≤ 8, and for a vortex beam with *m* > 8 we used a vortex retarder (VR). The vortex beam is directed *via* a mirror *M* into a high-NA objective O2 (100×, NA 1.25, Oil-immersion, Nikon Inc.), producing a tightly focused beam at the focal plane. PS nanoparticles (∼100 nm in diameter, 250 mg/10 mL) serve as probe particles to visualize the SOC effect. They are dispersed individually in deionized water (W119424-25L, Aladdin) at a volume ratio of 1:2000. The O2 was also utilized for imaging. For sample illumination, a white LED is positioned above the liquid surface, providing bright light for imaging through O1 (10×). To improve imaging clarity, a lens L3 (focal length of 200 mm) focuses the image onto a high-frame-rate CCD camera (1,280 × 1,024 pixels, DCC1240C-HQ, Thorlabs) connected to a computer. This arrangement allows the CCD camera to record particle motion in real-time, capturing their trajectories within the vortex field with high precision. Furthermore, the entire system is mounted on an optical shock-absorbing platform to minimize external vibrations. A semi-enclosed transparent box encloses the sample to avoid airflow disturbances from affecting the PS nanoparticles.

**Figure 4:**
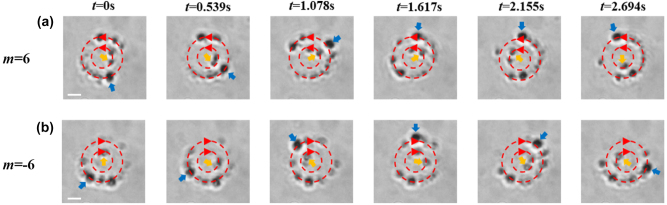
The dual-orbit motion of PS nanoparticles at a topological charge of |*m*| = 6. (a) and (b) Snapshots from video sequences recorded for at *m* = 6 (a) and *m* = −6 (b) illustrate the dual-orbit motion. The red dashed lines indicate the dual orbits, and the red arrows show the rotation direction of the particles. The scale bars in (a) and (b) each represent 2 µm.

To validate the flexible manipulation of nanoparticles using the SOC effect in the inverted optical tweezers platform shown in Figure 3, we employed a tightly focused vortex beam to perform precise dual-orbit rotational operations on PS nanoparticles, as illustrated in Figure 4. Figure 4 presents the orbital motion of multiple aggregates composed of PS nanoparticles along inner and outer orbits under topological charges *m* = 6 and −6, respectively. Here, the formation of these aggregates is due to the attractive van der Waals interactions at short distances between the 100 nm diameter PS particles in deionized water. Additionally, the minimum aggregate radius measured in the experiment is approximately 200 nm, attributed to the bright field microscopy and white-light illumination, where the diffraction limit generally restricts resolution to about 150–250 nm [5], [51]. The particles’ dual-orbit motion was recorded through video sequence snapshots, and their rotation velocities and directions were analyzed under opposite topological charges. For *m* = 6, Figure 4(a) shows a snapshot of the aggregates’ orbital motion (Supplementary Movie 1 for the dual-orbit counterclockwise rotation of multiple nanoparticles under *m* = 6). It is clear that the nanoparticle aggregates are confined by radial trapping forces to two distinct orbits: a smaller-radius inner orbit and a larger-radius outer orbit. These two orbits correspond to the central bright ring (for *m* = 6) and the adjacent secondary bright ring (for *m* = 8) of OAM density in Figure 1, respectively. In Figure 4(a), the red dashed lines denote the dual orbits, red arrows indicate the rotation direction, and yellow and blue arrows highlight the trajectories of tracked particles in the inner and outer orbits, respectively. Experimentally, the particles rotate steadily and uniformly counterclockwise, with average rotation velocities of 3.7 rad/s in the inner orbit and 1.2 rad/s in the outer orbit. The rotation velocity on the inner orbit is significantly higher than on the outer orbit, which is also predicted by the OAM density in Figure 1 and optical force in Figure 2. To assess the influence of topological charge sign on particle rotation direction, Figure 4(b) presents particle motion snapshots at *m* = −6 (see Supplementary Movie 2). For negative topological charges, the particles rotate clockwise along the orbits, opposite to the counterclockwise rotation observed at *m* = 6. The average rotation velocity is 4.2 rad/s in the inner orbit and 1.6 rad/s in the outer orbit.

**Figure 5:**
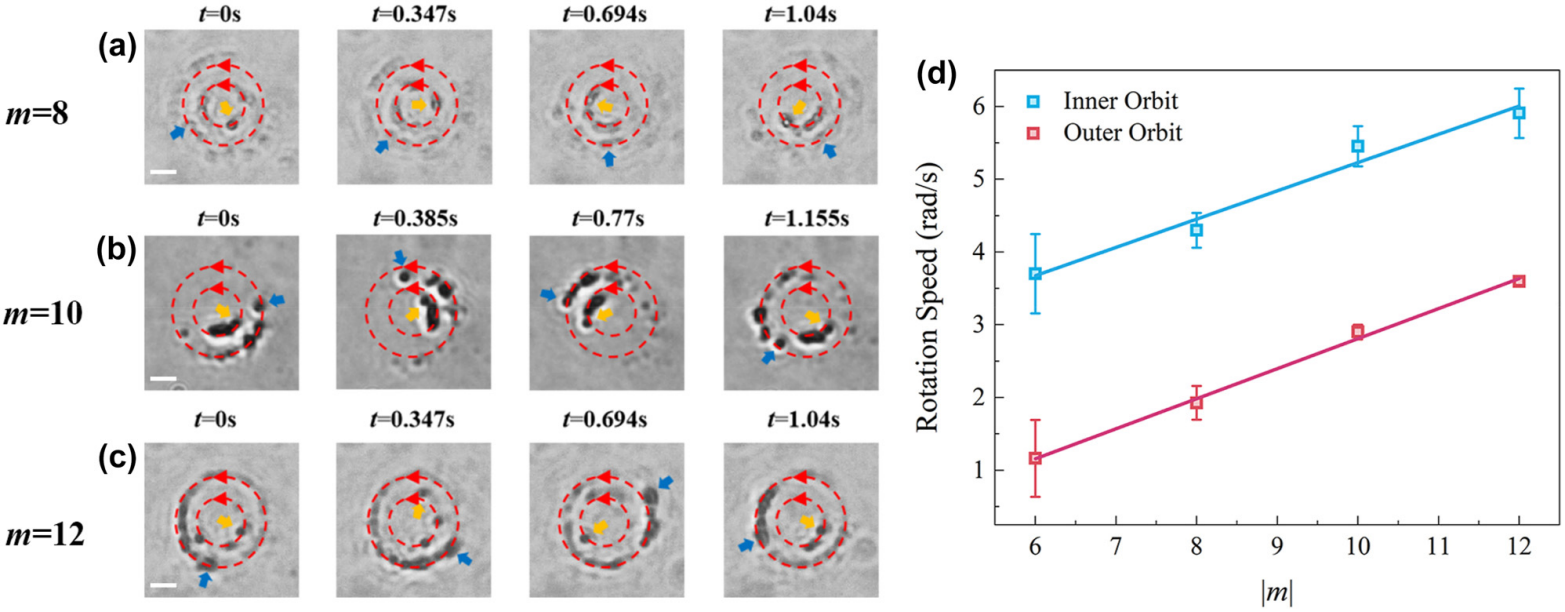
The dual-orbit motion of PS nanoparticles under different topological charges. (a)–(c) Snapshots from video sequences at different topological charge *m* = 8, 10, and 12, respectively. (d) The dependence of nanoparticle rotation velocity in the inner and outer orbits on various topological charges. The scale bars in (a), (b) and (c) each represent 2 µm.

The orbital rotation velocity of particles plays a crucial role in the optical manipulation process based on the SOC effect. As shown in Figures 1 and 2, theoretical calculations and numerical simulations indicate that as the topological charge increases, the tangential driving force *F*_*φ*_ for particles with a given size gradually increases. Consequently, the orbital rotation velocity of the particles also increases. To explore this relationship, the influence of topological charge magnitude on the particle rotation velocity was investigated, as shown in Figure 5. Figure 5(a)–(c) are snapshots of the orbital motion of the particle at *m* = 8, *m* = 10 and *m* = 12, respectively. When the topological charge is increased to *m* = 8, the particles continue to rotate stably in the counterclockwise direction with an increasing rotation velocity (see Supplementary Movie 3). The average rotation velocity on the inner orbit increases to 4.3 rad/s, while the outer orbit shows an average velocity of 1.9 rad/s. At *m* = 10, the rotation velocity increases, as illustrated in Figure 5(b) (see Supplementary Movie 4). With a further increase to *m* = 12, the average rotation velocity achieves 5.9 rad/s on the inner orbit and 3.6 rad/s on the outer orbit, as shown in Figure 5(c) (see Supplementary Movie 5). The rotation velocity increases further, indicating that the absolute value of the topological charge is positively correlated with the rotation velocity. Intriguingly, regardless of the topological charge magnitude, the rotation velocity on the inner orbit consistently exceeds that of the outer orbit. To quantify this relationship, Figure 5(d) shows the rotation velocities of particles on the inner and outer orbits for different topological charges. The results reveal a strong linear correlation between the topological charge and the rotation velocity, with correlation coefficients of 0.96 (inner orbit) and 0.99 (outer orbit), respectively. Simultaneously, the orbital radius of the particle’s rotation increases with the topological charge. For instance, when the topological charge *m* increases from 6 to 12, the inner orbit diameter grows from 3.64 μm to 4.15 μm, and the outer orbit diameter expands from 6.97 μm to 7.85 μm. This observation aligns with the theoretical prediction in Figure 1. In turn, by adjusting both the sign and magnitude of the topological charge, not only can the rotation direction of the particles be flexibly controlled, but their rotation velocity can also be regulated. Finally, we conducted experiments using PS particles with diameters of 900 nm and 2.5 μm to validate the influence of particle size on the detection of the dual-orbit dynamics induced by the SOC effect. Supplementary Movie 6 shows the clockwise dual-orbit motion of multiple 900 nm diameter PS particles at a topological charge of *m* = −6. As the particle diameter increases to 900 nm, the dual-orbit motion remains observable. However, because of the increased particle size, the gradient force acting on these particles intensifies, causing them to preferentially occupy the inner orbit, where the light intensity is higher. Additionally, as the particle size approaches the gap between the orbits, particles on both orbits begin to interact with each other. The particle on the outer orbit is affected by the gradient force from the inner orbit and moves into the inner orbit, while the particle on the inner orbit is pushed into the outer orbit. Supplementary Movie 7 shows the clockwise rotation of 2.5 μm diameter PS particles around the orbit when the topological charge is *m* = −6. In this case, when the particle size exceeds the orbit gap, the particles rotate around a single orbit and do not exhibit dual-orbit motion. Therefore, the selection of appropriately sized particles as probes for the visualization of the SOC effect is crucial. The proposed mechanism broadens the degrees of freedom and flexibility in the optical manipulation of micro-nano particles, with potential applications in optical sorting, self-assembly, and all-optical directional transport of particles.

## 4 Conclusions

In summary, a novel dual-orbit rotational dynamics of nanoparticles induced by the SOC effect in a tightly focused circularly polarized LG beam has been reported, combining theoretical analysis, numerical simulations, and experimental validation. Theoretical analysis indicates that the tightly focused effect facilitates the conversion from SAM to OAM, leading to a multi-ring structure in the OAM distribution. In particular, it is the central bright ring and its adjacent secondary bright rings that drive the dual-orbit motion of the nanoparticles. Numerical calculations of the normalized optical force further revealed its influence on particle trapping and rotation. In the experiments, the flexible manipulation of PS nanoparticles was achieved using the inverted optical tweezer system, thus verifying the feasibility and effectiveness of the proposed SOC effect. The results showed that the PS nanoparticles were stably confined to inner and outer orbits by radial trapping forces, and the tangential driving force compelled the particles to rotate around the beam axis. Importantly, by adjusting the magnitude and sign of the topological charge, the rotational velocity and direction of the particles could be precisely controlled, providing new insights and methods for future multi-degree-of-freedom manipulation techniques based on optical fields.

## Description of the Supplementary Movies

**Movie 1.** The video shows the counterclockwise dual-orbit motion of multiple aggregates composed of PS nanoparticles (∼100 nm) along inner and outer orbits under topological charges *m* = 6. The scale bar represents 2 μm.

**Movie 2.** The video shows the clockwise dual-orbit motion of nanoparticle aggregates under topological charges *m* = −6. The scale bar represents 2 μm.

**Movie 3.** The video shows the counterclockwise dual-orbit motion of nanoparticle aggregates under topological charges *m* = 8. The scale bar represents 2 μm.

**Movie 4.** The video shows the counterclockwise dual-orbit motion of nanoparticle aggregates under topological charges *m* = 10. The scale bar represents 2 μm.

**Movie 5.** The video shows the counterclockwise dual-orbit motion of nanoparticle aggregates at a topological charge *m* = 12. The scale bar represents 2 μm.

**Movie 6.** The video shows the clockwise dual-orbit motion of 900 nm diameter PS particles at a topological charge *m* = −6. The scale bar represents 2 μm. As the particle size approaches the gap between the orbits, particles on the two orbits begin to interact with each other.

**Movie 7.** The video shows the clockwise single-orbit motion of 2.5 μm diameter PS particles at a topological charge of *m* = −6. The scale bar represents 2 μm. Due to the particle size exceeds the orbit gap, the particles rotate around a single orbit and do not exhibit dual-orbit motion.
